# Viable *Mycobacterium tuberculosis* in sputum after pulmonary tuberculosis cure

**DOI:** 10.1186/s12879-019-4561-7

**Published:** 2019-10-30

**Authors:** Atiqa Ambreen, Muhammad Jamil, Mohammad Aqeel ur Rahman, Tehmina Mustafa

**Affiliations:** 1 Department of Microbiology, Gulab Devi Hospital, Lahore, Pakistan; 2 Department of Tuberculosis and Chest Medicine, Gulab Devi Hospital, Lahore, Pakistan; 3 Centre for International Health, Department of Global Public Health and Primary Care, University of Bergen, P.O. Box 7804, N-5020 Bergen, Norway; 4 Department of Thoracic medicine, Haukeland University Hospital, Bergen, Norway

**Keywords:** Pulmonary tuberculosis, Treatment response, Acid fast smear, Sterilizing cure, Ethambutol continuation phase

## Abstract

**Background:**

Pulmonary tuberculosis (TB) with detectable *Mycobacterium tuberculosis* in the sputum is a major source of transmission. In resource limited TB endemic settings, cure is declared through sputum smear examination for acid fast bacilli without performing culture. This may lead to erroneous treatment outcomes as viable bacteria may be missed due to the low sensitivity of direct smear method. The aim of this study was to investigate if sterilizing cure is achieved among the new pulmonary TB cases declared cured by sputum smear conversion and to evaluate the impact of addition of ethambutol in the continuation phase in achieving it.

**Methods:**

New sputum smear-positive pulmonary TB patients registered at a tertiary care hospital in Pakistan from November 2013 to March 2014 were followed under standard Directly Observed Treatment Short Course strategy for 6 months. Half of these patients received ethambutol in addition to isoniazid and rifampicin in the continuation phase. Sputum specimens were examined on microscopy at 2 months and at the end of treatment. Sputa of patients with negative direct smear examination at the end of treatment were cultured.

**Results:**

Among 5746 TB suspects, 1595 were new sputum smear positive pulmonary TB cases, and 533 were registered at our hospital. Among these, 504 converted sputum negative at 2 months and 348 converted at the end of 6 months of treatment and were declared cured. Sputa of 204/348 patients were cultured, and 12/204 (6%) were culture-positive. Culture positivity at 6 months was not associated with bacterial load, smoking, diabetes, presence of cavities, history of contact with TB patients, age, sex, socioeconomic status, or addition of ethambutol in the continuation phase.

**Conclusion:**

Viable cultivable bacilli were detected in 6% of cured patients, which would have significant impact on the control of TB. This highlights the need for an inexpensive and accurate surrogate marker for culture as it is not feasible to perform culture in routine for monitoring treatment response in the low-resource settings. The treatment outcome did not improve by addition of ethambutol emphasizing the need to find the optimal duration of treatment for individual or carefully selected groups of patients.

## Background

Tuberculosis (TB) is a major global health concern. As per World Health Organization (WHO) TB report in 2018, the annual global incidence has not decreased below 10.0 million [1]. Pulmonary TB with detectable *Mycobacterium tuberculosis* (MTB) in the sputum is a major source of transmission, and therefore a focus of global TB Control strategies. Sputum smear microscopy for acid-fast bacilli (AFB) is a widely available, simple, and inexpensive tool for pulmonary TB diagnosis and treatment monitoring [2]. The standard treatment for TB comprises an intensive phase with isoniazid (INH), rifampicin, pyrazinamide, and ethambutol for 2 months, followed by a continuation phase that comprises the concomitant use of INH and rifampicin for another 4 months [3, 4]. This standard treatment period of 6 months was determined by acceptable rates of treatment failure and disease recurrence after discontinuation of chemotherapy and is considered effective for drug-susceptible TB [5]. Response to TB treatment is monitored by follow-up sputum smear microscopy at 2 months and 5 months [4, 6]. Diminishing numbers of AFB to smear-negative status during treatment is considered an indication of treatment success. According to WHO a patient whose sputum smear or culture was positive at the beginning of the treatment but who was smear- or culture-negative in the last month of treatment and on at least one previous occasion is declared cured at the end of treatment [4]. In resource limited settings, Mycobacterial culture is not routinely performed on sputum smear-positive cases for monitoring treatment response [4]. In the absence of culture a negative sputum smear during the last months of treatment is considered as cure. There is little information as to whether bacterial sterilization is achieved after 6 months of treatment in all cases [7] and whether sputum smear conversion is a satisfactory method for measuring sterilizing cure.

A minimum two effective drugs is necessary in the continuation phase of treatment to achieve successful treatment outcome and to prevent the emergence of multidrug resistant strains. A drug resistance survey in Pakistan in 2016 has shown 7% INH resistance among rifampicin sensitive cases [8]. This implies that about 7% of the pulmonary TB cases would be receiving only one effective drug in the continuation phase. This could contribute towards failure to achieve the sterilization and persistence of a small number of bacilli even after treatment and emergence of resistant strains. According to WHO, ethambutol can be added in continuation phase of patients with known or suspected high levels of INH resistance, but more evidence is needed to support this recommendation [4]. The aim of this study was to investigate if sterilizing cure is achieved among the new pulmonary TB cases declared cured by sputum smear conversion and the impact of addition of ethambutol in the continuation phase in achieving sterilizing cure.

## Methods

### Study setting and design

The study was conducted at Gulab Devi hospital (GDH), from November 2013 to March 2014. GDH is a private, not-for-profit, large, tertiary care hospital located in Lahore, the capital city of the country’s largest province. GDH is known for specialized TB care and presumptive and diagnosed TB patients are referred from various districts to GDH for consultation and /or treatment. Many patients after the diagnosis are referred back to TB clinics close to their residence for treatment.

This study was nested in another project to study recurrence rate of TB by addition of a third drug (ethambutol) and prolongation of continuation phase to 6 months (submitted for publication). New, sputum smear-positive, pulmonary TB patients, without a history of previous TB treatment, were included only if they had successful sputum smear conversion at the end of treatment and also if the culture was performed. All these patients were given standard anti-TB treatment as per WHO guidelines [4]. Rifampicin, INH, ethambutol and pyrazinamide were given for the initial 2 months (Intensive phase). After 2 months, patients were split into two groups. One group was given rifampicin and INH for 4 months (continuation phase), while for the other group, ethambutol was added during the continuation phase along with INH and rifampicin. Follow up smears were done at the second, fifth and sixth months of treatment. If sputum smear was found positive at the end of second month, examination was repeated at third month of treatment as well. Patients who were having negative sputum smear at the end of treatment were declared cured. These patients were enrolled in the study only if their sputa were sent for culture.

If the sputum smear of a patient was found to be positive at the fifth or sixth month of treatment, the case was declared as treatment failure, excluded from the study and referred to the laboratory for *Xpert MTB/RIF* assay.. In cases where MTB deoxyribonucleic acid was detected and rifampicin resistance was not shown, category 2 treatment was started according to the WHO guidelines [4]. Patients with rifampicin resistance were referred to the Programmatic Management of Drug-Resistant Tuberculosis site at GDH for further treatment and management.

### Clinical and laboratory investigations

Detailed clinical history of the patients was taken, including history of smoking and diabetes. Physical examination was performed. Baseline blood tests, human immunodeficiency virus (HIV) screening and chest X-ray were done for patients on their first visit. Two (spot and early morning) sputum specimens were taken and examined for the presence of AFB by Ziehl-Neelsen staining method and direct microscopic examination [9]. All investigations were done at GDH laboratory which is a quality assured laboratory participating in the external quality assurance system provided by the provincial and national reference laboratories of Pakistan. Two sputum samples were examined at the time of diagnosis, while a single sputum was examined at each follow-up visit and at the end of treatment. The definition of a new sputum smear-positive pulmonary TB case was based on the presence of at least one acid-fast bacillus in the sputum specimen. The microscopy results were graded as per the guidelines of the International Union Against Tuberculosis and Lung Disease [10]. Sputa from the smear-negative cases at the end of treatment were processed for cultures on Lowenstein-Jensen (LJ) medium. Sputa were decontaminated and concentrated and the deposit was inoculated on two slopes of LJ medium [9]. The reading of culture slopes was done weekly. LJ culture tubes were kept for a maximum of 8 weeks at 37 °C before declaring them negative. Positive cultures were reported as soon as growth was detected. Identification was based on the phenotypic appearance of colonies and acid-fastness on Ziehl-Neelsen stained smears. Confirmation of *M. tuberculosis* complex was done by inhibition of its growth in a medium containing 0.5 mg/ml of para- nitrobenzoic acid [11]. In case of contamination in inoculated tubes, or growth of AFB mixed with contaminants, specimen /culture was reprocessed using N-acetyl-L-cysteine-sodium hydroxide method at a final concentration of 2% sodium hydroxide [9].

### Statistical analysis

The data was entered into SPSS version 20 and cleaned for further analysis. Binary logistic regression analysis was carried out to identify factors associated with achieving sterilizing cure. A Fisher’s Exact test was done to show the difference between the proportions in each treatment group. *P*-value of less than 0.05 was considered statistically significant.

## Results

### Patient characteristics

**Fig. 1:**
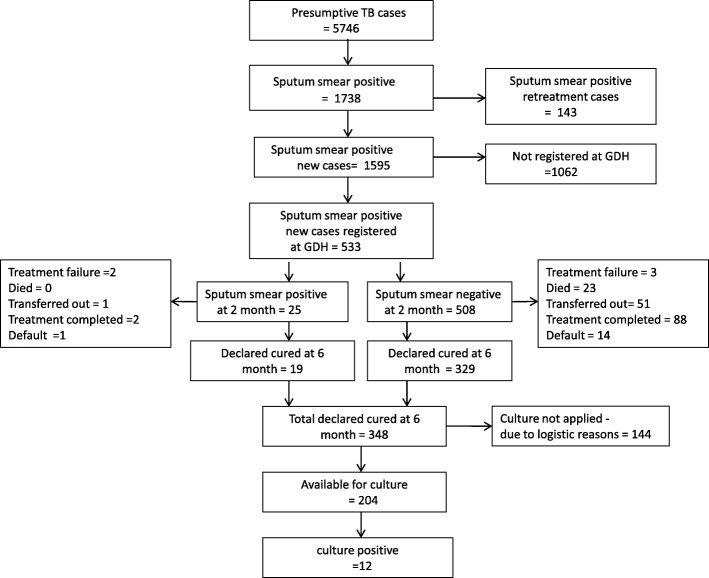
Flow chart of study design. GDH = Gulab Devi hospital, TB = Tuberculosis

A total of 5746 patients were identified as presumptive TB cases from November 2013 to March 2014 (Fig. 1). Among these 1738 (30%) were smear-positive for AFB, and 1595 (92%) were new TB patients who were never treated for TB before, or had taken anti-TB drugs for less than 1 month. Among these 533 (34%) were registered at GDH. After 6 months of treatment, 348 (65%) patients were declared cured by sputum smear microscopy. Culture was performed on 204 of these patients. The demographic and clinical data of these 204 patients with available culture results is shown in Table 1. Relatively few (6.8%) patients were smokers with a male predominance. The prevalence of diabetes mellitus was low (5.4%), and none of these patients were positive for HIV. Majority of the chest radiographs showed non-cavitary pulmonary infiltrates (93.1%), and cavities were seen in only 6.9% of cases. More than half of the patients belonged to low-income groups. History of TB contact from a household member was reported by 39% of the patients.

**Table 1:** Demographic and clinical characteristics of sputum smear-positive pulmonary TB patients

Age in years, median, (range)	25 (16–76)
Sex n (%)	
Male	94 (46)
Female	110 (54)
History of contact with TB, n/N (%)	
^a^ Recent	41/204 (20.0)
^b^ Previous	39/204 (19.2)
No history of contact	124/204 (60.8)
Prevalence of diabetes n/N (%)	11/204 (5.4)
Males	5/94 (5.3)
Females	6/110 (5.5)
Prevalence of HIV	0/204 (0)
Smokers n/N (%)	14/204 (6.8)
Males, n/N (%)	13/94 (13.8)
Females, n/N (%)	1/110 (0.9)
Chest X-ray	
Cavitary lesion, n/N (%)	7/102 (6.9)
Non-cavitary infiltrates, n/N (%)	95/102 (93.1)
Not available	102/204 (50.0)
Income Group (PKR/month)	
Low (0–15,000) n (%)	109 (53.4)
Middle (15,000 - 30,000) n (%)	9 (4.5)
Unknown n (%)	86 (42.1)

*n* number, *N* Total number, % percentage

^a^History of contact with active TB case during the course of diagnosis and treatment

^b^History of contact with active TB case before diagnosis and treatment

### Treatment outcomes

After 2 months of treatment, sputa from 201/204 (98.5%) patients converted negative. At the end of 6 months of treatment, sputa from all the patients had converted negative. However, 12 of these sputum smear-negative cases had viable bacteria recovered on their culture. Culture positivity at 6 months was not associated with bacterial load, smoking, diabetes, presence of cavities, history of contact with TB case, age, sex, socioeconomic status, or the additional drug in the continuation phase (Table 2).

**Table 2:** Factors associated with positive sputum culture after 6 months of anti-TB treatment

	Sputum status at the end of anti-TB treatment			^a^*P*-value
	Smear negative n/N (%)	Culture negative n/N (%)	Culture positive n/N (%)	
Age, years				
16–35	144/144 (100)	136/144 (94.4)	8/144 (5.6)	.708
36–55	44/44 (100)	40/44 ((90.9)	4/44 (9.1)	.999
56–76	16/16 (100)	16/16 (100)	0/16 (0)	.998
Male	94/94 (100)	88/94 (93.6)	6/94 (6.3)	.585
Female	109/109 (100)	103/109 (93.5)	6/109 (5.5)	
Smoker	14/204 (6.8)	14/14 (100)	0/14 (0)	.999
Diabetes	11/204 (5.3)	11/11 (100)	0/11 (0)	.999
^b^Bacillary load before treatment				
Scanty	20/204 (9.8)	18/20 (90.0)	2/20 (10.0)	.661
+ 1	117/204 (57.4)	109/117 (93.2)	8/117 (6.8)	.382
+ 2	37/204 (18.1)	36/37 (97.3)	1/37 (2.7)	.661
+ 3	30/204 (14.7)	29/30 (96.7)	1/30 (3.3)	.815
Smear positivity at 2 M	3/204 (1.5)	3/3 (100)	0/3 (0)	1.00
Chest X-ray				
Cavitary lesions	7/102 (6.9)	6/7 (85.7)	1/7 (14.3)	.265
Non-cavitary infiltrates	95/102 (93.1)	91/95 (95.8)	4/95 (4.2)	
History of contact				
^c^Recent	41/204 (20.0)	36/41 (87.8)	5/41 (12.2)	.08
^d^Previous	39/204 (19.2)	39/39 (100)	0/39 (100)	.998
No history of contact	124/204 (60.8)	119/124 (95.9)	5/124 (4.1)	.897
Income				
Low	109/204 (53.4)	102/109 (93.6)	7/109 (6.4)	.985
Middle	9/204 (4.5)	9/9 (100)	0/9 (0)	.862
Unknown	86/204 (42.1)	81/86 (94.2)	5/86 (5.8)	.999
Medicines in the continuation Phase				
INH + Rifampicin	103/204	4/103 (3.9)	99/103 (96.1)	.177
INH+ Rifampicin +Ethambutol	101/204	8/101 (7.9)	93/101 (92.1)	

*n* number, *N* Total number, % percentage

^a^Difference between culture positive and culture negative groups

^b^Graded as per the guidelines of the International Union Against Tuberculosis and Lung Disease

^c^History of contact with active TB case during the course of diagnosis and treatment

^d^History of contact with active TB case before diagnosis and treatment

### Treatment outcomes with additional drug in the continuation phase

Characteristics of patients receiving different drug regimens in the continuation phase were not different statistically (Table 3). Patients who were given additional ethambutol in the continuation phase had similar bacillary load and radiographic findings at the start of treatment as those who received the standard two drugs. The addition of ethambutol did not seem to have an impact on sputum sterilization at the end of treatment (*p* > 0.05).

**Table 3:** Characteristics of patients receiving different drug regimens in the continuation phase

	INH + Rifampicin	INH + Rifampicin + ethambutol
Age in years, median, (range)	25 (16–75)	25 (16–76)
Sex, n/N (%)		
Male	46/103 (44.6)	48/101 (47.5)
Female	57/103 (55.3)	53/101 (52.4)
History of contact with TB, n/N (%)		
^a^ Recent	21/103 (20.3)	20/101 (19.8)
^b^ Previous	17/103 (16.5)	22/101 (21.7)
No history of contact	65/103 (63.1)	59/101 (58.4)
Prevalence of HIV	0/103	0/103
Smoker n/N (%)	4/103 (3.8)	10/101 (9.9)
Prevalence of diabetes n/N (%)	8/103 (7.7)	3/101 (2.9)
^c^Bacillary load before treatment n/N (%)		
scanty	10/103 (9.7)	10/101 (9.9)
+ 1	57/103 (55.4)	60/101 (59.4)
+ 2	25/103 (24.2)	12/101 (11.9)
+ 3	11/103 (10.7)	19/101 (18.8)
Chest X-ray n/N (%)		
Cavitary lesions	3/7 (42.9)	4/7 (57.1)
Non-cavitary infiltrates	49/95 (51.6)	46/95 (48.4)
Not available	50/102 (49.1)	52/102 (50.9)

*n* number, *N* Total number, % percentage

^a^History of contact with active TB case during the course of diagnosis and treatment

^b^History of contact with active TB case before diagnosis and treatment

^c^Graded as per guidelines of the International Union Against Tuberculosis and Lung Disease

## Discussion

Standard 6 months of rifampicin containing treatment is considered curative for new drug-sensitive pulmonary TB, and the sputum smear conversion is considered as a reliable marker for successful treatment. In this study, we show that sterilizing cure was not achieved in all the cured patients after standard treatment, and viable cultivable bacilli were detected in 6% of patients despite successful sputum smear conversion. Earlier studies have shown that MTB may persist in lung tissue for months to years even after bacterial sterilization is achieved at the end of treatment [12–15]. A study has shown the presence of MTB mRNA in the context of non-resolving and intensifying lesions on positron emission tomography-computed tomography (PET–CT) images after treatment completion and bacterial sterilization suggesting that even apparently sterilizing curative treatment for TB may not eradicate all the MTB bacteria in most of the patients [7]. The persisting bacilli could lead to relapse as shown in a study from Uganda where 10% of successfully treated patients with standard 6 months regimen patients got recurrence of TB within 1 year, and 81% of these recurrent TB cases were due to relapse [16]. The relapse rates range from 2.6 to 9.7% after successful standard treatment and sterilization cure [13, 16–18]. Based on these findings it can be speculated that the relapse rate after non-sterilizing cure might be even higher, which would have a significant impact on the global control of TB. A study from Japan reported complete resolution of all active pulmonary TB lesions on PET-CT scan after 12 months of treatment, and no relapse at 1 year of follow-up [19]. These findings raise questions on the presence of viable bacilli after treatment and whether relapse is related to the duration of treatment. The higher relapse rates in patient groups with impaired immunity support the concept that a competent immune response has an important complementary role in the ultimate control of residual bacteria after completion of antibiotic treatment [18, 20, 21]. The findings of our study highlights the need for improvement in monitoring treatment response by developing a sensitive, specific and inexpensive surrogate marker for quantifying TB other than the gold standard of culture, as it is not feasible to perform culture in routine for monitoring treatment response in the low-resource high TB endemic setting.

Previous studies have shown that presence of the lung cavities on chest X-ray at the start of treatment is inversely proportional to sterilization at the end of treatment [22]. However, in our study, no association was seen with cavities at the start of treatment and sputum sterilization at 6 months of treatment. Culture positivity of sputum at 2 months is thought to be associated with culture positivity at 6 months implying high bacterial load at the start of treatment could lead to unfavorable outcomes [23]. We did not apply culture at 2 months but higher bacillary load at the start of treatment, or smear positivity at 2 months was not associated with culture positivity at the end of treatment. Lack of these associations could be due to a small number of patients with unfavorable outcome in our study.

In this study 5/12 culture-positive cases had a recent history of TB in their family, implying the possibility of reinfection from the home environment rather than treatment failure as the source of culture positivity. Different studies in the past have shown that exogenous reinfection could be a major cause of recurrent TB after achieving cure especially in TB endemic areas [24]. Patients who have had TB once are at an increased risk of developing TB when re-infected. Relapse is shown to occur early after treatment completion, whereas reinfection dominates after 1 year and shown to account for at least half of recurrent disease [25]. These findings emphasize the importance of achieving sterilizing cures and preventing transmission.

In our cohort, the proportion (5%) of diabetics among the TB patients was much lower than the prevalence of diabetes mellitus in the general population in Pakistan, which is shown to be 26.3% [26]. Additionally the lack of association of diabetes with culture positivity at the end of treatment does not confirm the earlier studies indicating diabetes as a risk factor for active pulmonary TB and unfavorable treatment outcomes [20, 27, 28]. The prevalence of diabetes in our study could have been underestimated as prevlance was based on patient history and serum glucose levels were not measured, leaving the possibility that some patients might have undiagnosed diabetes. A cross-sectional study from Lahore, Pakistan found 14.8% diabetics among TB patients by measuring serum glucose and HbA_1_C levels [29]. This prevalence is higher than our cohort but still lower than the prevalence in the general population. A Malaysian study reviewed 1267 active TB patients at a tertiary hospital and did not find diabetes as a risk factor for treatment failure [30]. Thus diabetes may not be a substantial risk factor for all pulmonary TB and unfavorable treatment responses.

Inadequate chemotherapy causing exposure of bacilli to a single effective drug can lead to selection of resistant subpopulations, and unfavorable treatment outcomes [31–33]. The national prevalence of 7% INH resistance among rifampicin sensitive cases in Pakistan implies that a substantial number of TB patients receive only one effective drug in the continuation phase [8]. However, in our study, addition of ethambutol in the continuation phase did not affect sterilization of sputum. Ethambutol is a bacteriostatic drug while INH is bactericidal. Despite WHO recommendation, the evidence to quantify the ability of ethambutol to achieve better outcomes when used in combination to “protect rifampicin” in patients with pretreatment INH resistance, is insufficient, and further research is needed.

There are some shortcomings in the study. It is a relatively small study, with a small number of unfavorable outcomes. Culture was not performed in all smear-negative cases due to resource constrains. Chest X-ray results could not be retrieved for several patients, and data on the recurrence of TB was not available. This could have added bias in the study. Despite these shortcomings, the study provides useful information about the treatment outcomes in the routine TB control program settings and could contribute towards evidence for improving routine TB care.

## Conclusions

This study shows that 6% of the pulmonary TB cases which were declared cured on sputum-smear conversion at 6 months did not achieve sterilizing cure and were not truly cured. These findings, extrapolated globally, would have significant impact on the control of TB. This emphasizes the need for an inexpensive and accurate surrogate marker for culture as it is not feasible to perform culture in routine for monitoring treatment response in the low-resource high TB endemic setting. Addition of ethambutol in the continuation phase did not result in a better sterilizing cure emphasizing the need for more studies to find the optimal duration of treatment, and adjusting TB treatment for individual or carefully selected groups of patients.

## Data Availability

The datasets used and/or analyzed during the current study are available from the corresponding author on reasonable request.

## Abbreviations

AFB: Acid-fast bacilli
CT: Computed tomography
GDH: Gulab Devi hospital
HIV: Human immunodeficiency virus
INH: Isoniazid
MTB: *Mycobacterium tuberculosis*
PET: Positron emission tomography
TB: Tuberculosis
WHO: World Health Organization
